# Metacognition, symptoms and general functioning in patients with schizophrenia

**DOI:** 10.1192/j.eurpsy.2021.1413

**Published:** 2021-08-13

**Authors:** M. Daoud, J. Ben Thabet, M. Maalej Bouali, S. Omri, R. Feki, N. Smaoui, L. Zouari, N. Charfi, M. Maalej

**Affiliations:** 1 Department Of Psychiatry “c”, Universty Hospital of Hedi Chaker, sfax, Tunisia; 2 Psychiatry C Department, Hedi chaker University hospital, sfax, Tunisia

**Keywords:** General function, metacognition, schizophrénia, Symptoms

## Abstract

**Introduction:**

Poorer metacognitive abilities are recognized as strong predictors of social functioning deficits in individuals with schizophrenia.

**Objectives:**

The aim of the current study is to examine metacognitive functioning in people with schizophrenia and to explore correlations between metacognition, symptoms and general functioning.

**Methods:**

It was a cross-sectional study involving outpatients diagnosed with schizophrenia and followed in the psychiatry “C” department at Hedi Chaker university Hospital, in Sfax -Tunisia, between may and december 2018. Sociodemographic, clinical and therapeutic data were measured using self-reported questionnaires, and metacognition was assessed with the Metacognition Assessment Scale – Abbreviated version (MAS-A). The general functioning was measured with The Global Assessment of Functioning (GAF).

**Results:**

A total of 74 participants participated in the study. The average age was 34.1 ± 11.8 years and the sex-ratio was 1.6. The average score of global assessment of functioning was 49.39±10. Means and standard deviations on MAS scores were as follows: self-reflectivity 4.18 (1.46), understanding of others’ minds 3.20 (1.06), decentration 2.5 (1.8), mastery 2.54 (1.85), and the MAS total scores 12.42 (6.17). The results indicate that poor social functioning is associated with metacognitive difficulties (r=0.27, p<10^-3^). Greater metacognition was significantly correlated with fewer negative symptoms (r= -0.62, p<10^-3^), but metacognition was not significantly correlated with positive psychotic symptoms, cognitive disorganisation, excitement or emotional distress

**Conclusions:**

These findings underscore the importance of interventions designed to enhance the patients’ metacognitive capacities, that is, the more proximal capacities linked to poorer social functioning.

